# Advances in IPMN imaging: deep learning-enhanced HASTE improves lesion assessment

**DOI:** 10.1007/s00330-025-11857-x

**Published:** 2025-07-21

**Authors:** Johannes Kolck, Fabio Pivetta, Clarissa Hosse, Haoyin Cao, Uli Fehrenbach, Thomas Malinka, Moritz Wagner, Thula Walter-Rittel, Dominik Geisel

**Affiliations:** 1 Charité—Universitätsmedizin Berlin, Department of Radiology, Berlin, Germany; 2 Charité—Universitätsmedizin Berlin, Department of Surgery CCM/CVK, Berlin, Germany

**Keywords:** Intraductal papillary mucinous neoplasms (IPMN), Deep learning, Half-Fourier single-shot turbo spin-echo (HASTE), Magnetic resonance imaging (MRI), Diagnostic confidence

## Abstract

**Objective:**

The prevalence of asymptomatic pancreatic cysts is increasing due to advances in imaging techniques. Among these, intraductal papillary mucinous neoplasms (IPMNs) are most common, with potential for malignant transformation, often necessitating close follow-up. This study evaluates novel MRI techniques for the assessment of IPMN.

**Materials and methods:**

From May to December 2023, 59 patients undergoing abdominal MRI were retrospectively enrolled. Examinations were conducted on 3-Tesla scanners using a Deep-Learning Accelerated Half-Fourier Single-Shot Turbo Spin-Echo (HASTE_DL_) and standard HASTE (HASTE_S_) sequence. Two readers assessed minimum detectable lesion size and lesion-to-parenchyma contrast quantitatively, and qualitative assessments focused on image quality. Statistical analyses included the Wilcoxon signed-rank and chi-squared tests.

**Results:**

HASTE_DL_ demonstrated superior overall image quality (*p* < 0.001), with higher sharpness and contrast ratings (*p* < 0.001, *p* = 0.112). HASTE_DL_ showed enhanced conspicuity of IPMN (*p* < 0.001) and lymph nodes (*p* < 0.001), with more frequent visualization of IPMN communication with the pancreatic duct (*p* < 0.001). Visualization of complex features (dilated pancreatic duct, septa, and mural nodules) was superior in HASTE_DL_ (*p* < 0.001). The minimum detectable cyst size was significantly smaller for HASTE_DL_ (4.17 mm ± 3.00 vs. 5.51 mm ± 4.75; *p* < 0.001). Inter-reader agreement was for (*к* 0.936) for HASTE_DL_, slightly lower (*к* 0.885) for HASTE_S_.

**Conclusion:**

HASTE_DL_ in IPMN imaging provides superior image quality and significantly reduced scan times. Given the increasing prevalence of IPMN and the ensuing clinical need for fast and precise imaging, HASTE_DL_ improves the availability and quality of patient care.

**Key Points:**

***Question*** *Are there advantages of deep-learning-accelerated MRI in imaging and assessing intraductal papillary mucinous neoplasms (IPMN)?*

***Findings*** *Deep-Learning Accelerated Half-Fourier Single-Shot Turbo Spin-Echo (HASTE*_*DL*_) *demonstrated superior image quality, improved conspicuity of “worrisome features” and detection of smaller cysts, with significantly reduced scan times.*

***Clinical relevance*** *HASTEDL provides faster, high-quality MRI imaging, enabling improved diagnostic accuracy and timely risk stratification for IPMN, potentially enhancing patient care and addressing the growing clinical demand for efficient imaging of IPMN.*

**Graphical Abstract:**

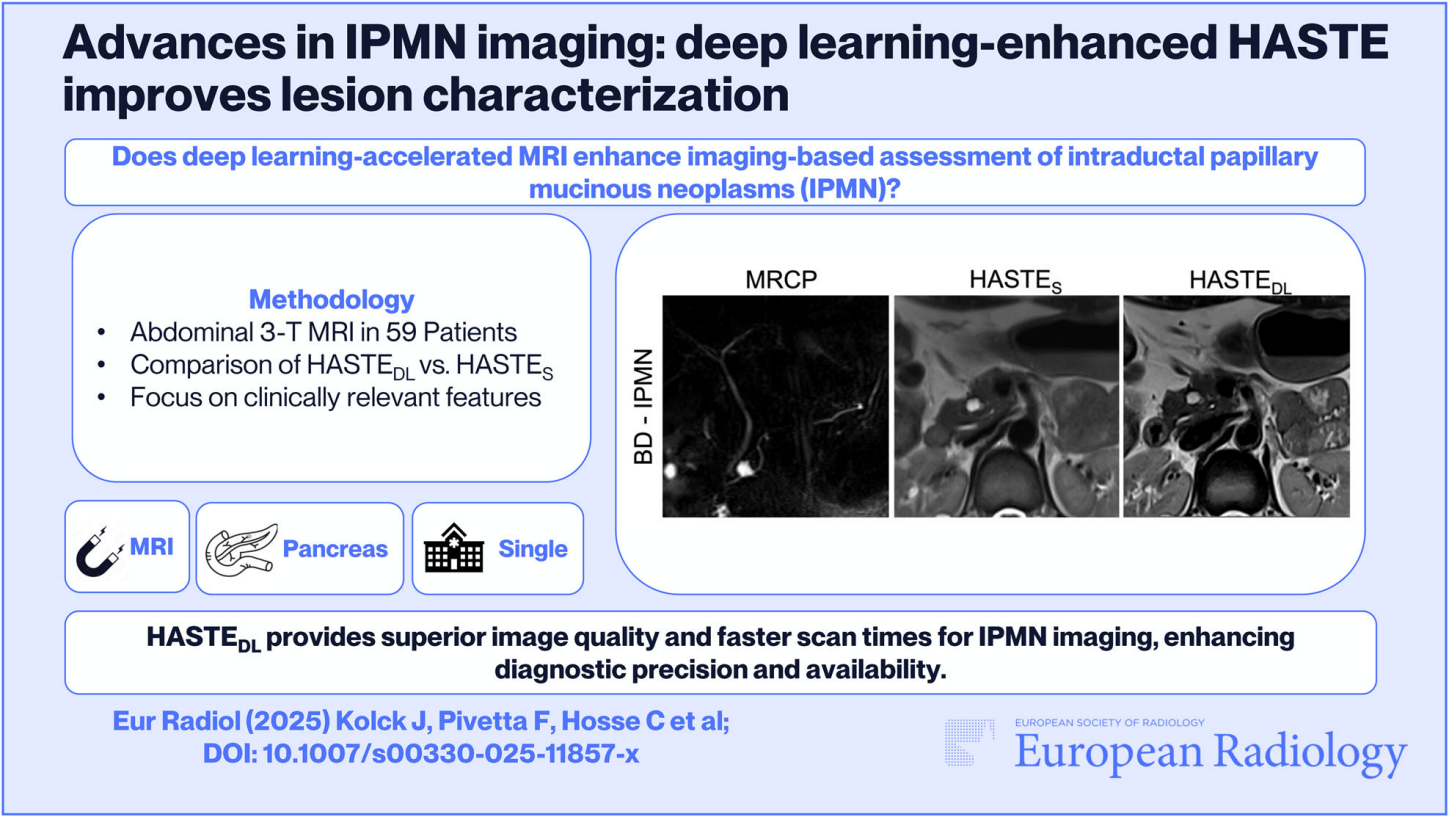

## Introduction

The prevalence of asymptomatic pancreatic cysts is estimated at 8% [1]. However, due to the widespread utilization of cross-sectional imaging and continuous enhancements in image quality, the detection of incidental pancreatic cysts has surged over recent decades. Up to 44.7% of magnetic resonance cholangiopancreatography (MRCP) scans reveal cystic pancreatic lesions [2]. Among these, intraductal papillary mucinous neoplasms (IPMNs) are particularly common. IPMNs can be categorized into three types based on imaging and/or histology: main Duct IPMN (MD-IPMN), branch duct IPMN (BD-IPMN), and mixed type [3]. Approximately 80% of cysts incidentally discovered are classified as BD-IPMNs [4, 5]. Although all IPMN subtypes carry a risk of malignant transformation, it is most pronounced in MD-IPMN, with rates of up to 70% [6, 7]. Risk stratification is based on “high-risk stigmata”, and “worrisome features”. While “high-risk features” are strongly suggestive of malignancy (e.g., obstructive jaundice, large nodules), “worrisome features” are indicators that raise concern but are less definitive. Apart from elevated carbohydrate antigen (CA) 19-9 levels, all “worrisome features” rely on imaging findings. These include cyst size ≥ 3 cm, enhancing mural nodules, thickened enhancing cyst walls, main pancreatic duct (MPD) diameter, abrupt change in MPD caliber with distal pancreatic atrophy, lymphadenopathy, and cyst growth of > 5 mm in 2 years or 2.5 mm per year, respectively [8–14].

Management of BD-IPMN has evolved in recent years, shifting from early surgical intervention to the more cautious, observation-focused approach [15]. Currently, surveillance intervals and duration remain a topic of debate [16]. Japanese studies highlight the risk of pancreatic cancer even in BD-IPMN smaller than 5 mm [17]. Consequently, the recent Kyoto guidelines suggest lifelong surveillance [16]. The decision for surveillance, especially in older patients, should consider the patient’s health status, comorbidities, life expectancy, and preferences to balance risks [16].

The high prevalence of BD-IPMN and the central role of imaging in classification and follow-up underscore the need for fast and precise imaging. To this end, our study sought to evaluate both qualitative and quantitative effectiveness of a Deep-Learning Half-Fourier Single-shot Turbo Spin-Echo (HASTE_DL_) sequence compared to a standard HASTE (HASTE_S_) at 3T-MRI of BD-IPMN.

## Materials and methods

### Study design and patient population

The study was approved by the Institutional Review Board (Internal registration number: EA4/195/24) and adhered to the principles outlined in the Declaration of Helsinki. Patient consent was waived by the ethics committee. Patients undergoing abdominal MRI between 1 May 2023 and 31 December 2023 with identified IPMN were retrospectively included in this study. Inclusion criteria included age over 18 years and confirmed presence of IPMN. Patients were excluded if only one MRI sequence (HASTE_DL_ and HASTE_S_) was available instead of both.

### Image acquisition

All examinations were conducted on clinical 3-Tesla (3-T) scanners (MAGNETOM Skyra and Vida; Siemens Healthcare) with patients in the supine position using an 18-channel body phased-array and a 32-channel spine phased-array coil. The MR imaging protocol for each patient included a conventional axial T2-weighted HASTE_S_ and HASTE_DL_ sequence with a single breath hold in axial orientation. Detailed sequence parameters are provided in Table 1.

**Table 1:** Sequence parameters for HASTE_DL_ and HASTE_S_

Parameters	HASTE_DL_	HASTE_S_
Orientation	Axial	Axial
Breath holds; interval (s)	1	3; 10 s
TE/TR (ms)	98/600	96/1600
Slice thickness (mm)	4	5
FA (°)	130-90-110-130	160
Slice gap (%)	20	17
Voxel size (mm³)	0.5 × 0.5 × 3.0	0.5 × 0.5 × 5.0
Acceleration factor	3	2
Acquisition time (min:s)	00:40	01:14

*HASTE*_*DL*_ Deep-Learning Half-Fourier Single-shot Turbo Spin-Echo, *HASTE*_*S*_ Standard Half-Fourier Single-shot Turbo Spin-Echo, *TE* echo time, *TR* repetition time, *FA* flip angle

### Image reconstruction with HASTE_DL_

The HASTE sequence with deep-learning (DL)-based reconstruction used in this study is a research tool. It incorporates a modified pulse sequence and reconstruction algorithm as previously described [18, 19]. It uses a regular k-space sampling scheme, similar to parallel imaging, with separate calibration data acquisition to generate coil sensitivity maps. To minimize crosstalk during short repetition time (TR) acquisitions, the slice increment between consecutive lines is set to four. In addition, variable flip angle evolution is implemented for refocusing pulses within the echo train. The DL reconstruction was based on a variational network that processes undersampled k-space data together with pre-computed coil sensitivity maps. This iterative approach alternates between parallel image-based data consistency updates and hierarchical neural network-based image enhancement. The network was trained offline in a supervised manner using approximately 10,000 slices from volunteers who provided written informed consent in accordance with local IRB guidelines. The resulting network parameters were then exported and integrated into the scanner’s prospective reconstruction pipeline by Siemens Healthineers.

### Image analysis

MRI images were evaluated using a Picture Archiving and Communication System (PACS) viewing station by two experienced radiologists (F.P. and J.K.) with more than 5 years’ experience in abdominal imaging. Each sequence was assessed independently, one month apart, to prevent potential recall bias.

### Quantitative and qualitative analysis

Quantitative image analysis evaluated the minimum detectable IPMN diameter and lesion contrast. The smallest detectable cystic pancreatic lesion identifiable as an IPMN was measured in mm. Image contrast between the pancreatic parenchyma and the largest identified lesion was assessed by placing a circular region of interest within the largest detected lesion and adjacent, healthy pancreatic tissue.

Qualitative analysis examined general sequence features, such as image quality, sharpness, contrast, and artifacts, as well as IPMN-specific factors, such as conspicuity, identification of complex features (dilated MPD, mural nodules, and septa), and detection of peripancreatic lymph nodes. All of these features were rated on a scale from 1 to 4 (1 = not detectable, 2 = poor detectability, 3 = good detectability, 4 = excellent detectability). The visualization of cyst communication with the MPD was rated as detectable or non-detectable.

### Statistics

Normally distributed variables are presented as median ± standard deviation (SD), non-normally distributed data are presented as median with interquartile range (IQR), while categorical variables are presented as frequencies (percentages). A paired Wilcoxon signed-rank test with continuity correction was used to compare the size of the smallest detectable lesion and the contrast between parenchyma and lesion. The chi-squared test was used to compare qualitative analysis results. Inter-reader agreement was assessed using Cohen’s kappa coefficient (*κ*). Statistical significance was set at *p* < 0.05. Statistical analyses were performed using SPSS software version 29.0.

## Results

### Study population

**Fig. 1:**
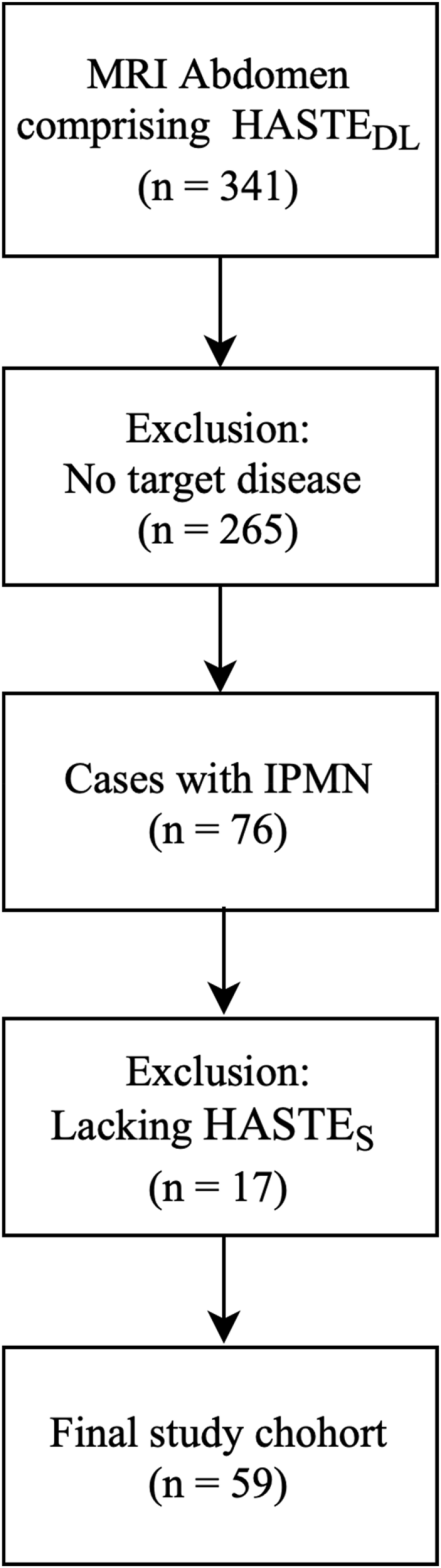
Flow chart of patient selection

A total of 341 MRI examinations of the abdomen incorporating the HASTE_DL_ sequence were obtained during May and December 2024. Among these, 76 patients were identified with IPMN. 59 of these underwent MRI examinations, including both HASTE sequences (Fig. 1). These 59 individuals (*n* = 26 female; *n* = 33 male) met the inclusion criteria and were enrolled. The mean patient age was 67 ± 11.62 years. The average height was 172.10 ± 10.48 cm, and the average weight was 72.0 (64.0–82.5) kg, resulting in an average body mass index (BMI) of 24.12 (22.28–27.05) kg/m². In most cases (67.8% or 40 patients), IPMNs were discovered incidentally. In 19 patients, underwent was performed as part of routine follow-up for previously diagnosed IPMN (Table 2).

**Table 2:** Overview of patient data and IPMN types

Age	67.88 ± 11.62
Height	172.10 ± 10.48
Weight	72.0 (64.0–82.5)
BMI	24.12 (22.28–27.05)
Incidental IPMN	67.80%
IPMN per patient	2.0 (1.0–3.5)
Complex features	45.76%
BD-IPMN	94.91%
MD-IPMN	1.69%
Mixed type	3.38%

*IPMN* intraductal papillary mucinous neoplasms, *BMI* body mass index, *BD-IPMN* branch duct intraductal papillary mucinous neoplasm, *MD-IPMN* main duct intraductal papillary mucinous neoplasm

### Inter-reader reliability

The inter-reader agreement between the two radiologists for the HASTE_DL_ was excellent (*к* 0.936), with particularly high agreement for lesion conspicuity (*к* 0.949) and visibility of a duct connection (*к* 0.983). Inter-reader agreement was good to excellent for HASTE_S_ (*к* 0.885), with the highest agreement in visibility of duct connection (*к* 0.966).

### Image quality

The overall image quality was highest for HASTE_DL_, with a median score of 3.90 ± 0.29, compared to a score of 3.24 ± 0.43 for HASTE_S_ (*p* < 0.001). Additionally, HASTE_DL_ received the highest ratings for sharpness and contrast with scores of 3.98 ± 0.09 and 3.97 ± 0.15, respectively, compared to 3.05 ± 0.25 and 3.27 ± 0.52 for HASTE_S_ (*p* < 0.001 and *p* = 0.112). Artifacts were scored with 3.5 ± 0.56 for HASTE_DL_, compared to 3.28 ± 0.55 for HASTE_S_ (*p* = 0.0019). These results are compiled in Table 3.

**Table 3:** Comparison of HASTE_S_ and HASTE_DL_ sequences for overall quality, sharpness, contrast, and artifacts, rated by two readers using a 4-point Likert scale with corresponding Cohen’s Kappa for inter-reader agreement

	HASTE_S_			HASTE_DL_		
	Reader 1	Reader 2	*Cohen’s K*	Reader 1	Reader 2	*Cohen’s K*
Overall quality	3 (3–3.5)	3 (3–3)	0.97	4 (4–4)	4 (4–4)	0.97
Sharpess	3 (3–3)	3 (3–3)	0.95	4 (4–4)	4 (4–4)	0.98
Contrast	3 (3–3.5)	3 (3–3)	0.97	4 (4–4)	4 (4–4)	0.98
Artifacts	3 (3–3.5)	3 (3–3)	0.93	4 (4–4)	4 (4–4)	0.97

*HASTE*_*DL*_ Deep-Learning Half-Fourier Single-shot Turbo Spin-Echo, *HASTE*_*S*_ Standard Half-Fourier Single-shot Turbo Spin-Echo

### Detectability and characterization of lesions

**Fig. 2:**
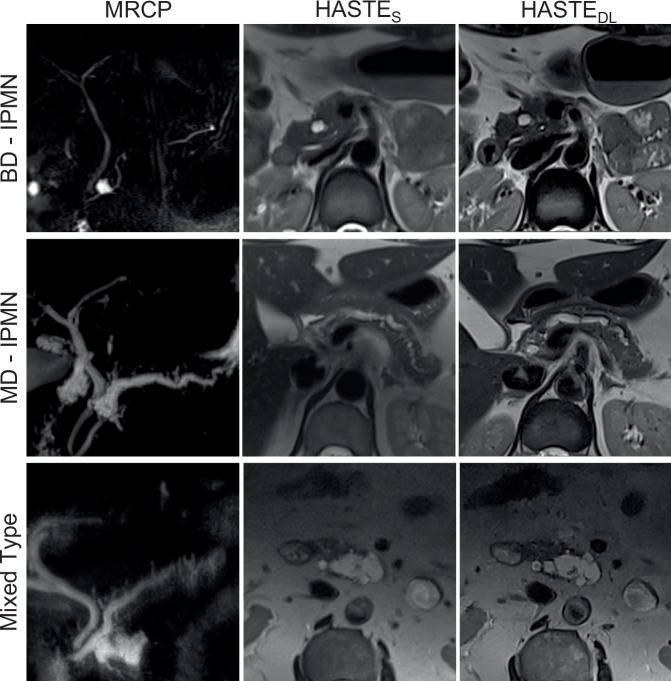
Overview of IPMN types, each shown with MRCP, HASTE_S_, and HASTE_DL_ sequences. Note the improved visualization of the communication with the main duct in BD-IPMN, the nodular component in MD-IPMN, and both septations and nodular component in the mixed-type IPMN when applying HASTE_DL_

**Fig. 3:**
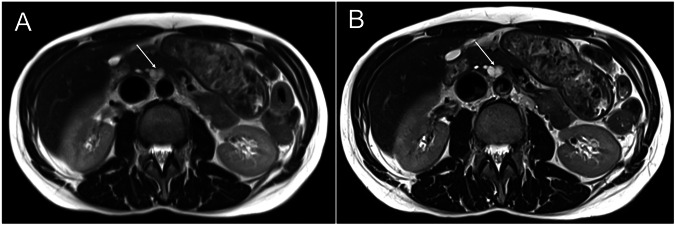
Comparison of HASTE sequences. Examination showing an IPMN with septa (arrow). **A** Standard HASTE. **B** Deep-Learning HASTE

**Fig. 4:**
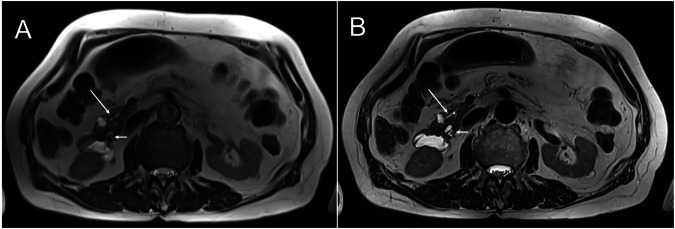
Comparison of HASTE Sequences. Examination showing two IPMNs in the pancreatic head. One with communication with the pancreatic duct (long arrow), and another with a mural nodule (short arrow). **A** Standard HASTE. **B** Deep-Learning HASTE

**Fig. 5:**
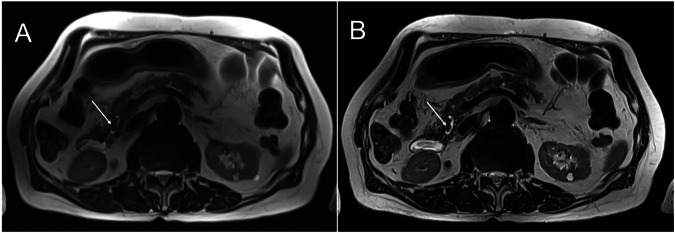
Comparison of HASTE Sequences. Examination showed an incidental calculus in the pancreatic duct. **A** standard HASTE. **B** Deep-Learning HASTE

Of the 59 enrolled patients, 32 had multiple IPMNs. There was one MD-IPMN and two mixed types, while all others were characterized as BD-IPMN. Detectability of IPMN was rated as good to excellent in both sequences, with significantly higher scores for HASTE_DL_ (mean score: 3.84 ± 0.33) compared to HASTE_S_ (mean score: 3.47 ± 0.56; *p* < 0.001). Communication of IPMN with the MPD was detected in 52.5% with HASTE_S_ vs. 71.2% with HASTE_DL_ (*p* < 0.001). Complex features, such as dilated MPD, mural nodules, and septa, were detected in 27 of 59 patients. Visualization of complex features was superior for HASTE_DL_ compared to HASTE_S_ (mean score: 1.94 ± 0.99 vs. 1.69 ± 0.79; *p* < 0.001). The presence of intralesional septa was the most common complex feature, identified in 20/59 (33.9%) cases. Similarly, detection of lymph nodes was superior for HASTE_DL_ vs. HASTE_S_ (mean score: 2.77 ± 0.54 vs. 1.87 ± 0.59; *p* < 0.001). There were no detectable lymph nodes in four patients (6.77%). Quantitative evaluations showed significant differences with respect to minimum detectable cyst size for HASTE_DL_ vs. HASTE_S_ (Ld_min = 4.17 mm ± 3.00 mm vs. Ld_min = 5.51 mm ± 4.75, *p* < 0.001). Contrast measurements were also superior for HASTE_DL_ compared to HASTE_S_ (*p* < 0.001). The data are summarized in Table 4, and Figs. 2, and 3–5 provide illustrative comparisons between HASTE_DL_ and HASTE_S_.

**Table 4:** Comparison of HASTE_S_ and HASTE_DL_ sequences for detectability, complex features, lymph nodes, and communication with the pancreatic duct, rated by two readers with corresponding Cohen’s Kappa for inter-reader agreement

	HASTE_S_			HASTE_DL_		
	Reader 1	Reader 2	*Cohen’s K*	Reader 1	Reader 2	*Cohen’s K*
Detectability	4 (3–4)	3 (3–4)	0.86	4 (4–4)	4 (4–4)	0.95
Complex features	1 (1–4)	1 (1–3)	0.86	1 (1–4)	2 (1–4)	0.92
Lymph nodes	2 (2–3)	2 (1–3)	0.85	3 (2.5–4)	3 (2–4)	0.90
Communication with the pancreatic duct	35%	33%	0.97	44%	43%	0.98

*HASTE*_*DL*_ Deep-Learning Half-Fourier Single-shot Turbo Spin-Echo, *HASTE*_*S*_ Standard Half-Fourier Single-shot Turbo Spin-Echo

## Discussion

This study aimed to evaluate the feasibility of a deep-learning-accelerated HASTE sequence (HASTE_DL_) for 3-T imaging of IPMN, in comparison to a clinically used conventional HASTE (HASTE_S_) sequence. Compared to HSATE_S_, HASTE_DL_ showed superior image quality, with enhanced detection of clinically relevant lesion features and improved visualization of smaller IPMN.

The prototypical HASTE_DL_ used in this study has been evaluated for abdominal and hepatic imaging in previous studies [14, 20, 21]. HASTE_DL_ had significantly shorter scan times and showed excellent image quality, only slightly inferior to T2-weighted Block Acquisition of Data in k-space sequence (BLADE) [14]. An inherent advantage of shorter scan times is the reduction of motion artifacts, which is particularly useful for patients who struggle with breath-holding [20, 21]. Two recently published studies investigated the performance of HASTE_DL_ in pancreatic imaging [22, 23]. In both studies, HASTE_DL_ outperformed BLADE and HASTE_S_ in qualitative and quantitative metrics, receiving higher scores for lesion conspicuity, reduction of motion artifacts, improvement of MPD visualization, overall image quality, as well as reduced intra-reader variability in lesion size measurements [22, 23].

To date, no study has specifically evaluated the utility of HASTE_DL_ for imaging IPMN, particularly with respect to clinically significant (“worrisome”) features, which are crucial for radiologic follow-up of these lesions [24]. Current imaging recommendations for the initial characterization of IPMN include breath-hold 2D or 3D axial in- and out-of-phase T1-weighted gradient-echo (GRE), axial and coronal half-Fourier single-shot fast spin echo (FSE) breath-hold T2-weighted, heavily T2-weighted 2D and/or 3D MRCP, as well as breath-hold or respiratory-navigated dynamic 3D fat-suppressed T1-weighted spoiled gradient echo (GRE) before and after IV gadolinium chelate contrast. Several studies have indicated that shorter protocols can be equally sufficient for follow-up imaging [25–27]. Following these observations, besides clear secondary benefits such as reducing examination time and lowering both the risk of contrast-related complications and financial costs, several societies have agreed on the recommendation of shortened protocols for follow-up imaging [28, 29].

However, most pancreatic cysts, and thus IPMN, are incidental findings on clinically indicated cross-sectional imaging [2], making initial assessment more challenging, as typical abdominal imaging protocols may not always include all of the recommended sequences but typically include a conventional HASTE sequence. Our study demonstrates that the use of HASTE_DL_ can improve risk stratification for incidentally detected IPMN by providing high-resolution, high-contrast imaging with reduced motion artifacts. In turn, improving conspicuity of septa, thickened cyst walls, mural nodularity, and peripancreatic lymph nodes. Each of these findings can represent a “worrisome feature” and may warrant further workup for sufficient decision making [8–13]. Moreover, HASTE_DL_ outperformed HASTE_S_ in the visualization of communication with the pancreatic duct, an essential imaging feature that aids in differentiating IPMN from serous cystic neoplasm (SCN) [30]. While higher MRI field strength is associated with improved anatomical visualization, it also increases the likelihood of artifacts [31]. Notably, in our study, no artifacts were observed that compromised diagnostic reliability. Apart from the diagnostic advantages described, HASTE_DL_ also significantly reduces scan times and increases signal-to-noise ratio (SNR), a distinct advantage over HASTE_S_, which has known limitations such as SNR and blurring due to the extended echo train length. The DL algorithms used in this study significantly improve the inherent limitations of HASTE_S_ and allow for a more precise evaluation of complex features of IPMN, also resulting in a higher inter-reader agreement.

Our study has several limitations. First, although we studied an elderly patient group, who face greater challenges and could benefit significantly from faster acquisition times, our sample size is relatively small. Additionally, the inclusion of only two readers and the single-vendor, single-center design limits the generalizability of our findings. Using different slice thicknesses for HASTE_DL_ (4 mm) and HASTE_S_ (5 mm) is a potential bias and confounder in our study. Thinner slices may inherently improve lesion conspicuity and spatial resolution, independent of the effects of DL reconstruction. Another limitation is that our study only included 3-T scanners; hence, the performance of HASTE_DL_ for IPMN imaging with 1.5-T scanners remains undetermined.

In conclusion, our results demonstrate the clinical feasibility of HASTE_DL_ in the detection and evaluation of IPMN by providing higher image quality along with significantly reduced scan times compared to HASTE_S_. Given the ever-growing patient population diagnosed with IPMN and the need for MRI imaging to classify and follow up on these lesions, HASTE_DL_ can help to improve access to imaging and lesion characterization.

## Abbreviations

BD-IPMN: Branch duct intraductal papillary mucinous neoplasm
BLADE: Block Acquisition of Data in *k*-space
DL: Deep learning
HASTE_DL_: Deep-Learning Half-Fourier Single-shot Turbo Spin-Echo
HASTE_S_: Standard Half-Fourier Single-shot Turbo Spin-Echo
IPMN: Intraductal papillary mucinous neoplasms
MD-IPMN: Main duct intraductal papillary mucinous neoplasm
MRCP: Magnetic resonance cholangiopancreatography
MRI: Magnetic resonance imaging
SNR: Signal-to-noise ratio
TE: Echo time
TR: Repetition time
*Κ*: Cohen’s Kappa coefficient
